# Therapeutic approach in Class I malocclusion with impacted maxillary canines

**DOI:** 10.1590/2177-6709.27.2.e22bbo2

**Published:** 2022-05-23

**Authors:** Matheus Melo PITHON

**Affiliations:** 1 Universidade Estadual do Sudoeste da Bahia, Departamento de Saúde I (Jequié/BA, Brazil).; 2 Universidade Federal do Rio de Janeiro, Programa de Pós-Graduação em Odontopediatria e Ortodontia (Rio de Janeiro/RJ, Brazil).

**Keywords:** Impacted maxillary canine, Corrective treatment, Traction

## Abstract

**Objective:**

To emphasize the importance of diagnosis and discuss the therapeutic approaches that can be used in the orthodontic treatment of Class I malocclusion associated with two impacted maxillary canines. The opening of spaces for traction of these teeth by means of rapid maxillary expansion or extraction of maxillary premolars was contraindicated in the case reported. Therefore, it was decided to open spaces with projection of incisors.

**Results:**

The obtained results were satisfactory, as a good occlusion was obtained, with adequate functional guides, as well as an improvement in the facial appearance.

**Conclusion:**

The projection of the incisors prior to traction of the impacted maxillary canines proved to be a valid option in the case described. Ten years after completion of treatment, the case is stable, maintaining periodontal health.

## RESUMO

**Objetivo:**

Destacar a importância do diagnóstico e discutir as abordagens terapêuticas que podem ser utilizadas no tratamento ortodôntico da má oclusão de Classe I associada a dois caninos superiores impactados.

**Relato de caso:**

A abertura de espaço para tracionar esses dentes por meio de expansão rápida da maxila ou exodontia de pré-molares superiores era contraindicada no caso relatado. Portanto, decidiu-se abrir espaço por meio da projeção dos incisivos.

**Resultados:**

Os resultados obtidos foram satisfatórios, pois obteve-se uma boa oclusão, com guias funcionais adequadas, além da melhoria no aspecto facial.

**Conclusão:**

A projeção dos incisivos prévia ao tracionamento dos caninos superiores impactados mostrou-se uma opção válida no caso descrito. Dez anos após a finalização do tratamento, o caso apresenta-se estável, mantendo a saúde periodontal.

## INTRODUCTION

Orthodontic traction of impacted canines is one of the greatest challenges in orthodontics.^1^ It is a relatively frequent clinical condition (0.9 - 2.2%),^2^ and treatment sometimes should include a multidisciplinary approach.^3^ Different etiologic factors are associated with impacted maxillary canines, such as ectopic location of the tooth germ, lack of space, lack of guidance, or genetic factors.^4^

Surgical exposure of the impacted canine and complex orthodontic mechanics applied to align the tooth in the respective arch can often lead to supporting tissue complications,^5^ in addition to a long treatment period and high costs for the patient. Therefore, early diagnosis is very important so that the problem can be managed as soon and efficiently as possible.^3^

Before implementing any type of traction, it is important to create space for the impacted tooth.^6^ This space can be created by maxillary expansion,^7^ dental projection,^3^ distalization of posterior teeth or extraction of permanent teeth adjacent to the impacted canine. The selection of the best treatment method rests upon correct diagnosis, to prevent adverse side effects.^8^

When the chosen option is the projection of anterior teeth, one cannot rule out the possibility of orthodontic treatment promoting the development of gingival recessions^9^ ^,^ ^10^ as orthodontic tooth movement could result in root positions that are close to or out of the cortical alveolar plates, leading to bone dehiscence.^11^ As a result, a marginal gingiva without appropriate alveolar bone support can migrate apically, thus exposing the root.^12^

The aim of the present study is to describe the orthodontic treatment of a Class I malocclusion associated with traction of two impacted canines for which the space created for their traction led to maxillary incisor proclination. 

## DIAGNOSIS

**Figure 1:**
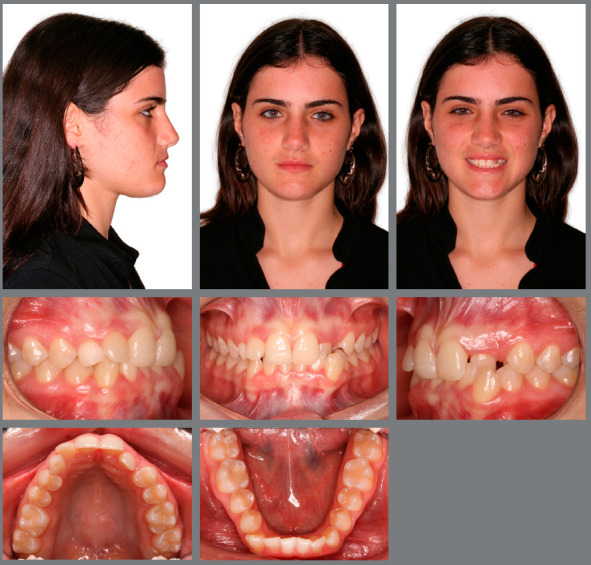
Pretreatment photographs.

The patient, aged 18 years and 10 months, sought orthodontic treatment with the following complaint: *“I have a milk tooth on one side, and on the other side, it goes inward into the arch.”* The patient’s general health status was good, and her medical history was unremarkable; presenting good oral health and oral hygiene; presence of a deciduous tooth (deciduous maxillary right canine); and onychophagia. Prior to the treatment, a full orthodontic documentation and computed tomography were requested for assessment of the missing teeth (Fig 1).

**Figure 2:**
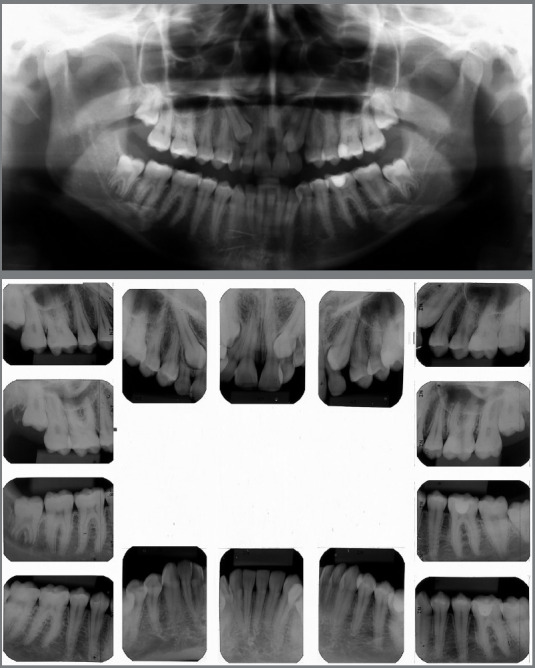
Pretreatment radiographs.

**Figure 3:**
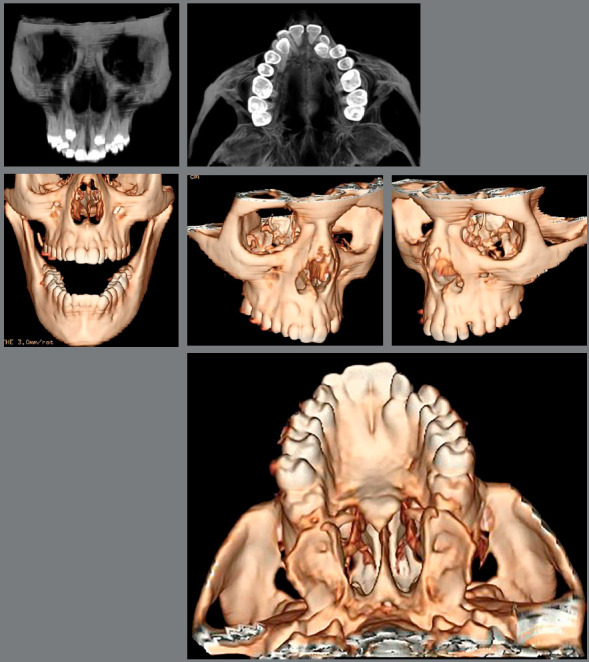
Pretreatment tomography.

**Figure 4:**
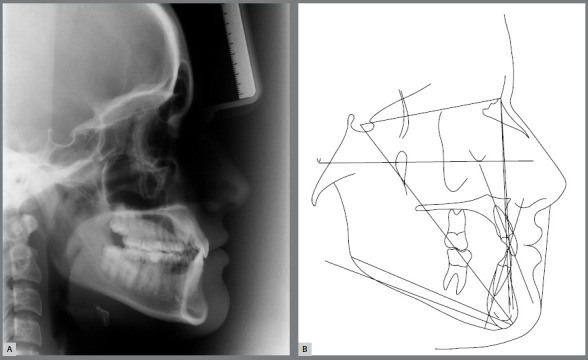
Pretreatment cephalometric radiograph and tracing.

**Figure 5:**
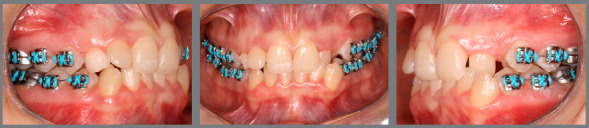
Alignment and leveling in the anterior region using segmented archwires.

The following facial features were observed: concave profile (upper lip-S line = -2 mm, lower lip-S line = -2 mm); passive lip seal; satisfactory nasolabial angle; short lower third of face; and unattractive gingival smile line, due to the presence of a deciduous tooth (deciduous maxillary right canine), missing teeth (permanent maxillary canines), and lingual crossbite (left maxillary lateral incisor). Clearly visible asymmetries were not detected. Malocclusion was classified as skeletal Class III (ANB = -10), with good relationship of the maxilla to the skull base and proclined mandible (SNA = 82º and SNB = 83º). There was balanced growth pattern, with horizontal facial growth tendency (SN.GoGn = 31º) (Figs 2, 3, 4 and 5).

Malocclusion was classified as Angle Class I, 3-mm overjet, and 4-mm severe overbite, mandibular anterior crowding, with arch length discrepancy of -3.7 mm; Bolton discrepancy of mandibular teeth, with excess of 0.3 mm in the anterior region; coincident midlines. The arches had a trapezoid shape and lacked space for maxillary canines, and left maxillary lateral incisor showed lingual crossbite (Figs 1 and 2). 

The panoramic radiograph revealed the presence of all permanent teeth and one deciduous tooth (deciduous maxillary right canine), with mesially inclined impacted maxillary canines. Extensive restoration was observed on mandibular left first molar (Fig 3).

The periapical radiographs more clearly revealed the same findings as the panoramic radiograph. It was not possible, however, to assess the integrity of the maxillary lateral incisor roots, and a CT scan of the face was then requested. The CT scan revealed that the integrity of the maxillary lateral incisor roots was preserved, and that the right maxillary canine was located buccally to the right maxillary lateral incisor, whereas permanent maxillary left canine was located lingually to left maxillary lateral incisor (Figs 3 and 4).

The cephalograms showed retroclined maxillary and mandibular incisors (1.NA = 19º and 1.NB = 20º) and lingually inclined mandibular incisors (1-NB = 2 mm) (Table 1 and Fig 5).

**Table 1:** Initial (A) and final (B) cephalometric values and 10 years after the end of treatment (C).

		MEASUREMENTS	Normal	A	A1	B	A/B	C
Skeletal Pattern	SNA	(Steiner)	82°	82°	83°	84°	2	81°
	SNB	(Steiner)	80°	83°	83°	84°	1	82°
	ANB	(Steiner)	2°	-1°	0°	0°	1	-1°
	Convexity Angle	(Downs)	0°	-6°	-4°	-2°	4	
	Y axis	(Downs)	59°	52°	52°	53°	1	53°
	Facial Angle	(Downs)	87°	94°	95°	94°	0	94°
	SN.GoGn	(Steiner)	32°	31°	31°	30°	1	31°
	FMA	(Tweed)	25°	21°	20°	21°	0	21°
Dental pattern	IMPA	(Tweed)	90°	87°	102°	91°	4	91°
	1.NA (degrees)	(Steiner)	22°	19°	35°	29°	10	29°
	1-NA (mm)	(Steiner)	4 mm	5mm	8mm	7mm	2	7mm
	1.NB (degrees)	(Steiner)	25°	20°	36°	24°	4	25°
	1-NB (mm)	(Steiner)	4mm	2mm	6mm	5mm	3	3mm
	- Interincisal Angle	(Downs)	130°	140°	108°	127°	13	126°
	1-APog	(Ricketts)	1mm	2mm	3mm	3mm	1	3mm
Profile	Upper Lip - S Line	(Steiner)	0mm	-2.5mm	-1mm	-1.5mm	1	-1.5mm
	Lower Lip - S Line	(Steiner)	0mm	-2mm	1mm	0mm	2	0mm

OTHER MEASURES	A	A1	B	A/B
Intercanine distance	25	24	24.4	1.4
Intermolar distance	42.5	44.6	43.9	1.4

In the functional evaluation, there was an absence of lateral guides in canines and of the anterior guide.

### TREATMENT OBJECTIVES

» Creation of space in the arch for accommodating the teeth.
» Traction of the impacted maxillary canines, not allowing them to contact the roots of lateral incisors.
» Maintenance of the patient’s dental and periodontal health.
» Improvement of facial profile, by incisor projection.

### TREATMENT ALTERNATIVES

1. Surgically-assisted maxillary expansion.
2. Maxillary expansion using an expander supported by skeletal anchorage.
3. Extraction of the maxillary first premolars to create space for traction of the maxillary canines.
4. Projection of maxillary incisors to create space for traction of the impacted maxillary canines.

### TREATMENT PROGRESS

**Figure 6:**
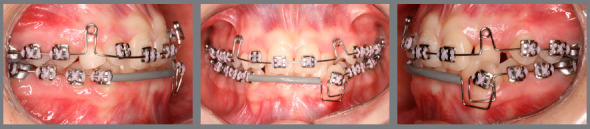
Initial projection of anterior teeth, combined with alignment and leveling of the mandibular arch using box loop mechanics.

**Figure 7:**
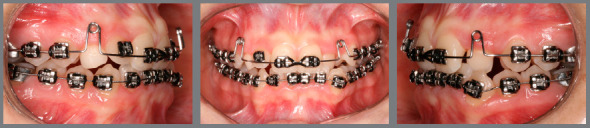
Intermediate phase of maxillary incisor projection.

**Figure 8:**
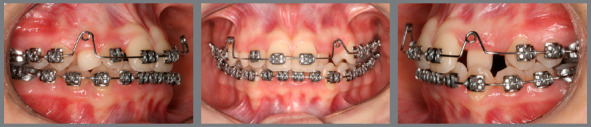
Final projection of maxillary incisors.

**Figure 9:**
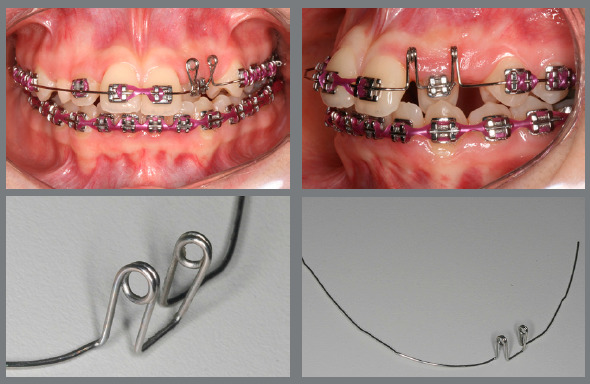
Maxillary archwire with loop for uncrossing the left maxillary lateral incisor.

**Figure 10:**
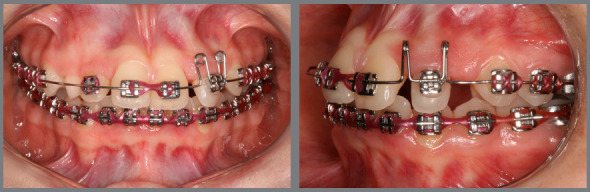
Left maxillary lateral incisor uncrossed after 27 days.

**Figure 11:**
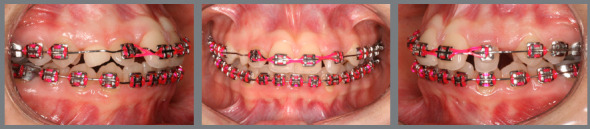
Mesial movement of maxillary lateral incisors using elastomeric chain.

**Figure 12:**
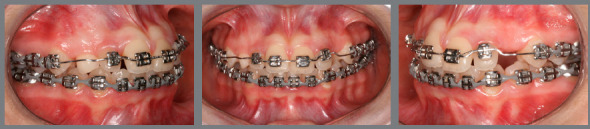
Space created for maxillary canines.

**Figure 13:**
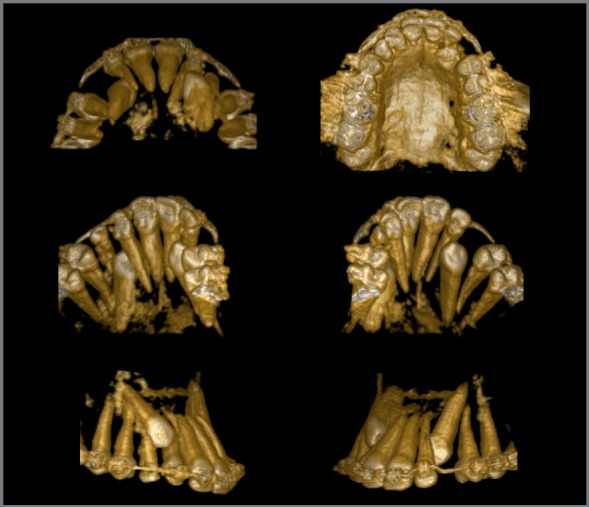
Tomography after incisor projection.

Based on clinical assessments and orthodontic records, a conservative orthodontic treatment was carried out (option 4), consisting of projection of maxillary and mandibular anterior teeth to create space for traction of maxillary canines (#13 and #23), and to allow alignment and leveling of teeth. The choice was made due to the impossibility of performing palatal disjunction and extraction of premolars. A fixed Edgewise standard appliance (0.022 x 0.028-in) was used. Bands were cemented in maxillary molars; simple brackets and tube for transpalatal arch were installed on the first molars and simple tubes on the second molars; and brackets were bonded to maxillary premolars. The following stainless steel segmented archwires were used for alignment and leveling: 0.015-in Twist Flex, 0.012-in, 0.014-in, 0.016-in, 0.018-in, and 0.020-in (Fig 5). Brackets were sequentially bonded to anterior teeth (maxillary right lateral incisor was bonded only for an esthetic purpose, without placing a wire in its slot), and 0.020-in stainless steel continuous passive archwire with double helical loops was installed on the mesial aspect of maxillary first premolars (this archwire was used to project the maxillary incisors), omega loops were used as a stop (Figs 6, 7 and 8). After space creation, crossed maxillary left lateral incisor was bonded, and a 0.018-in archwire with helical loops (buccal-palatal direction) was installed to uncross the maxillary left lateral incisor (Fig 9). After uncrossing the maxillary left lateral incisor, maxillary lateral incisors were mesially inclined with an elastomeric chain (Figs 10, 11 and 12). Thereafter, a 0.021 x 0.025-in passive stainless steel archwire was installed in association with wire ligature of the posterior and anterior teeth. Another CT scan was requested to assess the roots of the maxillary lateral incisors and the position of maxillary canines (Fig 13).

**Figure 14:**
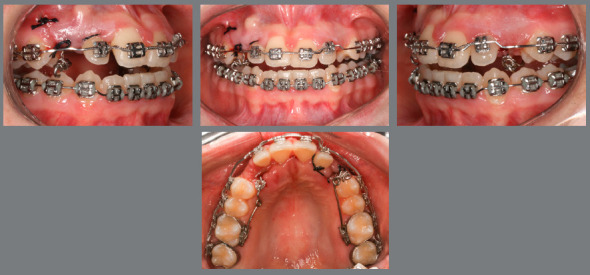
Shortly after intraoperative bonding of attachments on canines.

**Figure 15:**
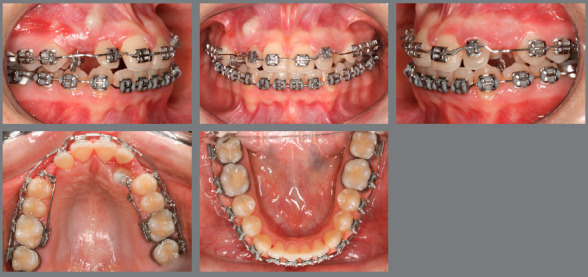
Traction of canines using a cantilever.

**Figure 16:**
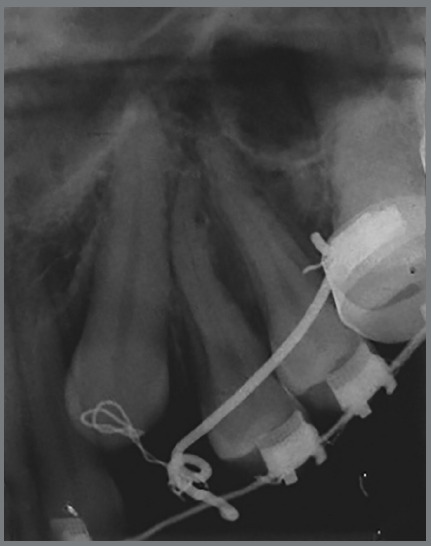
Periapical radiograph showing the line of action of the force.

**Figure 17:**
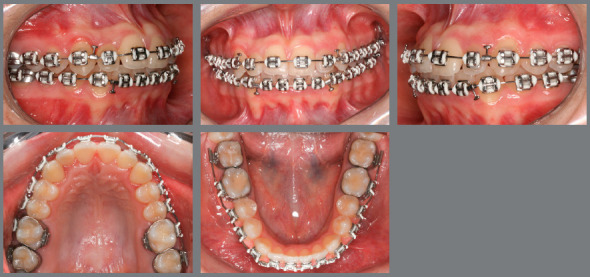
Orthodontic finishing after traction of the canines.

Extraction of maxillary deciduous right canine and intraoperative placement of the orthodontic device on the buccal aspect of maxillary right canine and maxillary left canine were requested (Fig 14). Ten days after the surgical procedure, the patient came back to the office and a cantilever device supported on the lingual tube of the maxillary first molars was installed for traction of maxillary right canine and left canine (Figs 15 and 16). With the full exposure of the crowns of maxillary right canine and left canine, the lingual buttons were removed, the brackets were bonded, a 0.012-in NiTi overlay archwire and a passive 0.021 x 0.025-in stainless steel archwire were fitted for alignment of those teeth (Fig 17).

**Figure 18:**
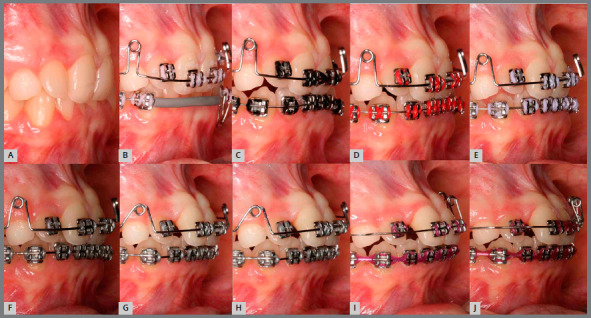
Sequential anterior space creation by tooth projection before traction ( right side ). A) baseline; B) initial projection; C) after 15 days; D) after 27 days; E) after 54 days; F) after 81 days; G) after 108 days; H) after 123 days; I) after 135 days; J) 162 days after projection.

**Figure 19:**
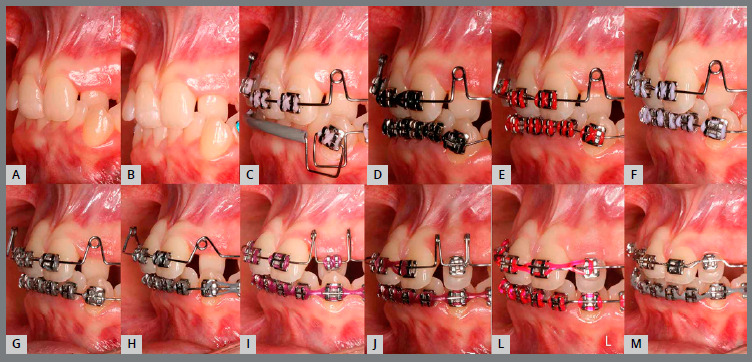
Sequential anterior space creation by tooth projection before traction ( left side ). A) baseline; B) posterior alignment and leveling; C) initial projection; D) after 15 days; E) after 27 days; F) after 54 days; G) after 81 days; H) after 108 days; I) after 123 days; J) after 135 days; L) 162 days after projection; M) space created prior to traction.

**Figure 20:**
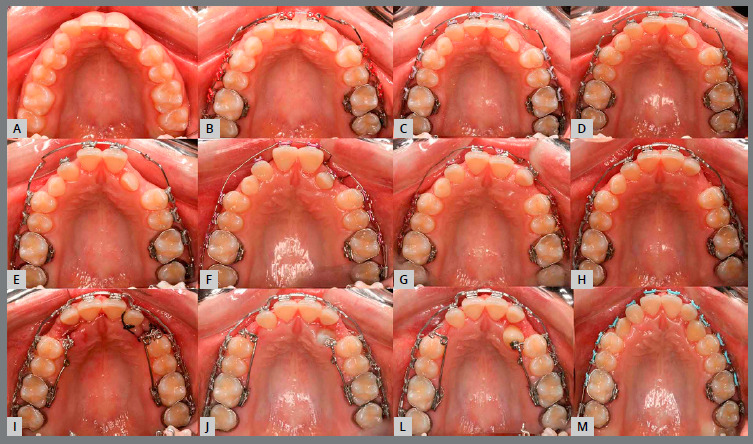
Sequential anterior space creation by tooth projection before traction ( upper arch ). A) baseline; B) initial projection; C) after 15 days; D) after 27 days; E) after 54 days; F) after 81 days; G) after 123 days; H) 162 days after projection; I) Intraoperative bonding of canines; J) initial traction; L) left maxillary canine subjected to traction and M) canines positioned in the arch.

**Figure 21:**
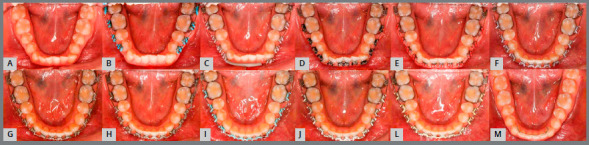
A) Baseline; B) initial alignment and leveling of posterior segment; C) continuous archwire with box loop for alignment of left mandibular canine; D) 0.015-in Twist-Flex; E) 0.012-in stainless steel ( SS ) archwire; F) 0.016-in SS archwire; G) 0.018-in SS archwire; H) 0.020-in SS archwire; I) 0.017 x 0.025-in SS archwire; J) 0.019 x 0.025-in SS archwire; L) archwire with finishing bends and M) end of treatment.

When the canines were closer to the line of occlusion, the maxillary dental arch was aligned and leveled again using coordinated and symmetrical 0.014-in, 0.016-in, 0.020-in, and 0.018 x 0.025-in steel archwires (Figs 18, 19, 20 and 21).

An elastomeric chain was placed along the archwire for closure of the remaining spaces, followed by installation of a 0.019 x 0.025-in steel finishing archwire, with ideal and coordinated shape and torque. Intermaxillary elastics for posterior (1/8-in heavy) and anterior (5/16-in medium) intercuspation were also used.

A fixed Edgewise standard appliance (0.022 x 0,028-in) was used in the mandibular arch (Fig 21). Bands were bonded and cemented to mandibular molars, with placement of simple brackets on the first molars and simple tube on the second molars and bracket placement on mandibular premolars. Posterior segmented stainless steel archwires (0.012-in, 0.014-in, 0.016-in, 0.018-in, and 0.020-in) were fitted for posterior alignment and leveling.

After that, bonding was carried out on canines and a 0.020-in archwire with a box loop was fitted for alignment and leveling of mandibular left canine. Bonding then proceeded on mandibular incisors and new 0.016-in, 0.018-in and 0.020-in archwires were fabricated for alignment and leveling of the teeth. A 0.019 x 0.026-in steel archwire with ideal and coordinated shape and torque was installed for treatment finishing. Intermaxillary elastics for posterior (1/8-in heavy, 8.8 oz) and anterior (5/16-in medium, 12.5 oz) intercuspation were also used.

After completion of the treatment, the orthodontic appliance was removed and a maxillary wraparound plate and mandibular fixed intercanine bar (3x3) were installed.

## TREATMENT OUTCOMES

**Figure 22:**
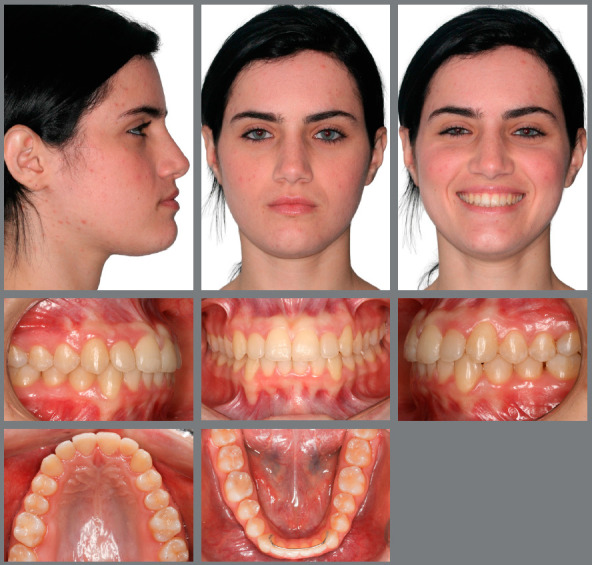
Post-treatment photographs.

**Figure 23:**
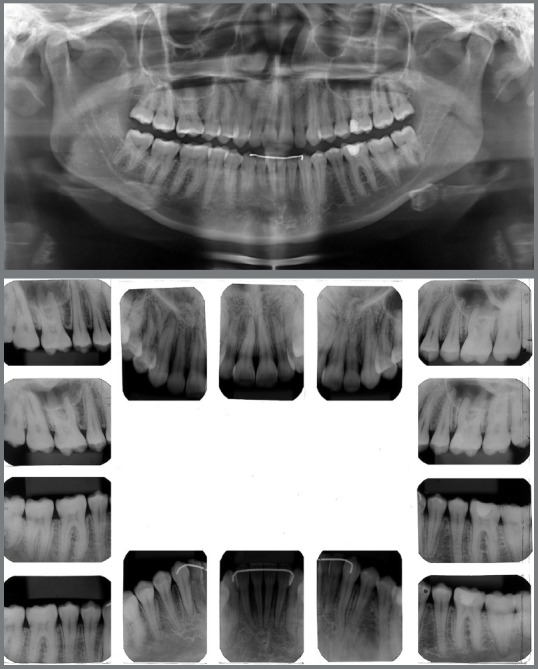
Post-treatment radiographs.

**Figure 24:**
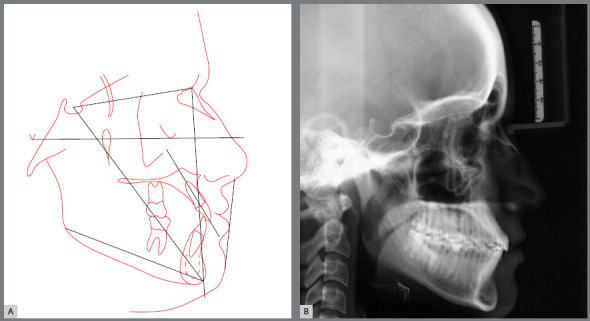
Post-treatment cephalometric radiograph and tracing.

The facial profile was straightened and passive lip seal and nasolabial angle were maintained. The features of the lower third of the face remained unchanged. The gingival smile line was improved after extraction of the deciduous tooth, tooth inclinations were also improved, and permanent canines were moved into their natural position (Figs 22, 23 and 24).

**Figure 25:**
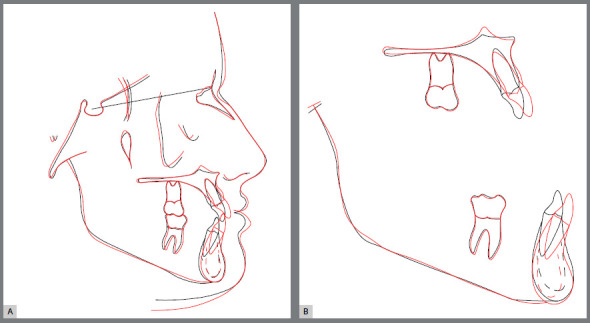
Superimposed cephalometric tracings.

The skeletal relationship between the maxilla and the mandible was enhanced, probably due to repositioning of point A, with maintenance of horizontal growth pattern (Figs 24 and 25).

Class I molar relationship was maintained; crowding in both dental arches was corrected; space was created in the arch for canines; maxillary canines were subjected to traction; the mandibular premolars and molars were uprighted; and the mesiodistal diameter of mandibular anterior teeth was adjusted, eliminating the existing Bolton discrepancy. The buccolingual position of maxillary left lateral incisor was corrected; contact points were improved; overjet and overbite were corrected; and the arches were harmonious and symmetrical, with maintenance of coincident midlines. The initial shape of the arches was maintained, and root and bone health was preserved.

Bilateral and simultaneous contacts were obtained, in harmony with centric relation and with posterior disocclusion and anterior guidance in mandibular excursions.

Superimposed cephalometric tracings revealed maintenance of vertical and anteroposterior position of the maxilla and mandible, and discrete projection of upper and lower lips. In partially superimposed cephalograms of the maxilla, maxillary incisors were projected and their roots were subjected to palatal torque, with better inclination in relation to their bone base, whereas maxillary molars kept their vertical and anteroposterior position. The partially superimposed cephalometric image of the mandible shows projection of the mandibular incisors and maintenance of vertical and anteroposterior position of the mandibular molars (Fig 25).

**Figure 26:**
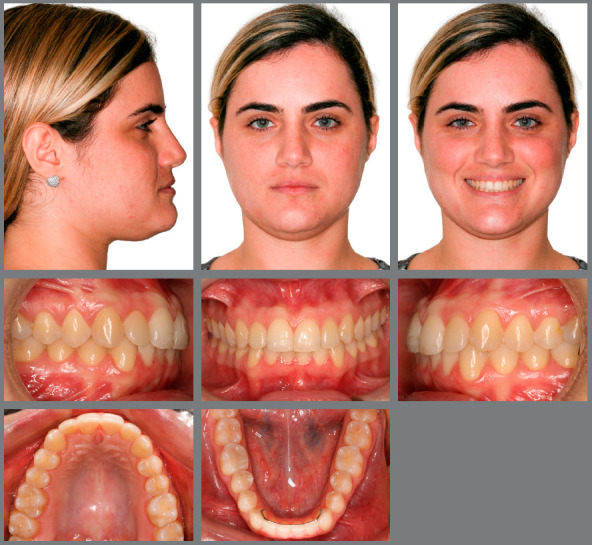
Photographs 10 years after treatment completion.

**Figure 27:**
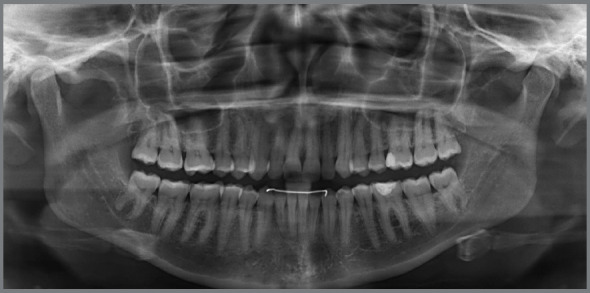
Panoramic radiograph 10 years after the end of treatment.

**Figure 28:**
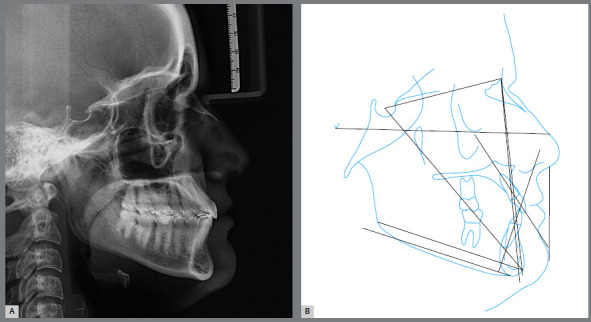
Cephalometric radiograph and tracing10 years after the end of treatment.

**Figure 29:**
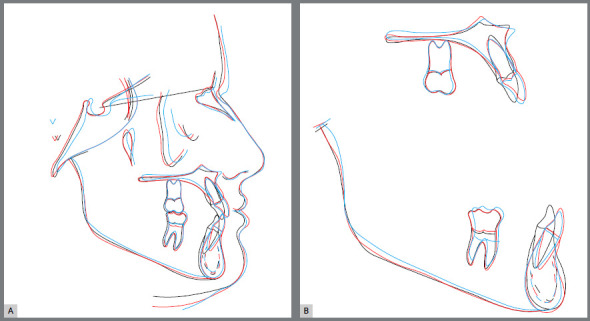
Superimposed cephalometric tracings.

Ten years after removal of the fixed orthodontic appliance, the improvements were maintained, as shown on the photographs and radiographs. Periodontal health was preserved without any periodontal involvement, despite projection of the anterior teeth to create space in the arches. The facial profile was more concave after 10 years due to residual mandibular and nose growth, observed with the reduction of the ANB angle (Figs 26, 27, 28 and 29).

## DISCUSSION

The permanent maxillary canines play a key role in shaping dentition and maintaining its function, and their presence in the dental arch is crucial for a balanced dynamic occlusion and for facial esthetics and harmony.^13^ ^-^ ^15^ Thus, a great deal of effort should be expended to maintain or to avoid the extraction of an impacted permanent maxillary canine^16^ ^-^ ^18^. The present clinical case illustrates very well this scenario, with two impacted maxillary canines, one buccally inclined and one palatally inclined, which were moved into the spaces created orthodontically.

The maxillary canine is the most frequently impacted tooth, in a palatal to buccal impaction ratio of 3:1 to 6:1.^19^ Girls are twice as often affected as boys,^20^ and bilateral impaction occurs in only 8% of the cases.^21^ The case described herein is therefore rare.

The first challenge in this case was the creation of enough space for the movement of canines. It has been widely described in the literature that creation of space is necessary prior to the orthodontic movement of any tooth.^6^ In the case of impacted canines, the creation of space often occurs via arch expansion or extraction of premolars.^7^ These approaches were not possible in the case described herein, as palatal disjunction was not indicated because the patient showed signs of calcification of the median palatine suture on the maxillary occlusal radiograph. Tooth extraction was also ruled out based on the patient’s facial pattern and concave profile. Tooth extraction in this situation eventually increases concavity, making patients look older than they are.^22^ Therefore, space was created by projecting the maxillary central incisors and extracting the right maxillary deciduous canine. The damage caused by tooth projection has been widely described in the literature.^23^ ^,^ ^24^ However, in the present case, the retroclined incisors and the concave facial profile favored the decision to project the teeth, which allowed for a more harmonious profile at the end of the treatment.

Another challenge was the close relationship between the lateral incisor roots and unerupted canines. In the presence of impacted teeth, especially canines, it is always important to request a CT scan^6^ ^,^ ^25^ to avoid surprises in the future. 

The canines responded well to traction, which did not cause any damage to the roots of lateral incisors closely related to their crowns or damage to the periodontal tissues of canines, which displayed good gingival insertion at the end of the treatment. The decision not to align and level the maxillary lateral incisors at the beginning of treatment prevented their roots from being moved against the maxillary canines. 

According to Yan et al.,^26^ physical proximity (< 1 mm) between the impacted canine and the adjacent root is the main predictor of root resorption. It should be highlighted that treatment of an impacted maxillary canine is not achieved exclusively by full orthodontic alignment. The final periodontal health is essential to assess the success of therapy, since biomechanical and surgical procedures can cause damage to the supporting tissues of the pulled and/or adjacent teeth.^27^

Precaution was taken during treatment regarding the traction of palatally impacted canines by subjecting keratinized tissue to traction, which prevents gingival recession.^28^ According to Ferreira et al.,^16^ knowledge of orthodontic mechanics is essential in cases of traction of impacted canines, and so is the management of applied forces.

Based on the records obtained at the end of the orthodontic treatment, it was possible to verify that all the proposed objectives were achieved. Class I relationship was obtained for molars and canines, satisfactory overjet and overbite, improvement in incisor inclination, alignment and leveling of all teeth, good periodontal health, intercuspation and adequate functional guides. The patient was satisfied with the results obtained mainly because she was informed at the beginning of the treatment that traction would be an attempt without guaranteed results.

## CONCLUSION

In conclusion, the projection of incisors for space creation prior to the traction of impacted maxillary canines is a feasible treatment option when the incisors exhibit good periodontal health.
